# Optimization of Deep Sedation with Spontaneous Respiration for Therapeutic Endoscopy Combining Propofol and Bispectral Index Monitoring

**DOI:** 10.1155/2015/282149

**Published:** 2015-08-13

**Authors:** Kohei Matsumoto, Akihito Nagahara, Kenshi Matsumoto, Yoichi Akazawa, Hiroyuki Komori, Yuta Nakagawa, Tsutomu Takeda, Hiroya Ueyama, Yuji Shimada, Daisuke Asaoka, Mariko Hojo, Sumio Watanabe

**Affiliations:** Department of Gastroenterology, Juntendo University School of Medicine, Tokyo 113-8421, Japan

## Abstract

*Background/Aims.* This study aimed to establish optimal propofol anesthesia for therapeutic endoscopy, which has not been established. *Methodology.* We retrospectively investigated data on 89 patients who underwent upper-GI endoscopic submucosal dissection or endoscopic mucosal resection under anesthesia with propofol. Examined doses of propofol were changed according to efficacy and/or adverse events and classified into 5 periods. A bispectral index (BIS) monitor was used at Period 5 to decrease the incidence of adverse events caused by oversedation. The initial dose of propofol was administered after bolus injection of pethidine hydrochloride (0.5 mg/kg), and 1.0 mL of propofol was added every minute until the patients fell asleep. Continuous and bolus infusion were performed to maintain sedation. When the patient moved or an adverse event occurred, the maintenance dose examined was increased or decreased by 5 mL/h regardless of body weight. *Results.* Dose combinations (introduction : maintenance) and patient numbers for each period were as follows: Period 1 (*n* = 27), 0.5 mg/kg : 5 mg/kg/h; Period 2 (*n* = 11), 0.33 mg/kg : 3.3 mg/kg/h; Period 3 (*n* = 7), 0.5 mg/kg : 3.3 mg/kg/h; Period 4 (*n* = 14), 0.5 mg/kg : 2.5 mg/kg/h; Period 5 (*n* = 30), 0.5 mg/kg : 2.5 mg/kg/h, using BIS monitor. During Period 5, an adverse event occurred in 10.0% of patients, which was lower than that for Periods 1–4. *Conclusions.* Period 5 propofol anesthesia with BIS protocol could be safe and useful for therapeutic endoscopy under deep sedation with spontaneous respiration.

## 1. Introduction

Therapeutic endoscopic procedures of increasing complexity have been developed in recent years. With the advent and widespread use of endoscopic submucosal dissection (ESD), indications for endoscopic treatment of early stage cancer of the digestive tract are expanding rapidly [1–3]. As a result, the number of therapeutic endoscopic procedures is increasing. However, for difficult lesions, potentially curative en bloc resection via ESD requires a lengthy procedure and advanced endoscopic skills, although it is less invasive than traditional surgical treatment. As ESD requires fine complicated maneuvers and any patient movement during the procedure can result in complications such as perforation, intraoperative management of the patient's general condition is very important to achieve safe ESD.

Benzodiazepines, such as diazepam and midazolam, have been used as a standard sedation in patients undergoing endoscopic therapy. However, a delay of several minutes occurs after the injection of benzodiazepines before an effect is exerted, and the range of effective doses of such agents differs considerably among patients, making it difficult to achieve a stable level of sedation. Also the dose is often increased to suppress body movements, leading to oversedation and potentially causing hypoxemia and decreased levels of consciousness after patients return to the hospital ward [4]. In addition, agitation occurs during the early stages of midazolam administration in rare cases [5].

The ideal agent for sedation during endoscopy would have the following characteristics: immediate onset, effects lasting only for the duration of the endoscopic procedure, prompt recovery from sedation without a period of residual mental or psychomotor impairment, and low frequency of sedation-associated side effects.

Propofol has an immediate onset (30–60 s) and half-life of 2.6 min, which is much shorter than those of benzodiazepine and is advantageous for induction and recovery from sedation. Guidelines on sedation using propofol have been published by the European Society of Gastrointestinal Endoscopy and the American Society of Anesthesiologists [6, 7]. Recently, several reports showed propofol to be superior to traditional sedative drugs because of its rapid onset of action and shorter recovery time [8–15]. On the other hand, propofol has a narrower safety range compared with other sedatives and presents risks of rapid depression of consciousness and cardiorespiratory function [16, 17].

In Japan, the safety instructions provided with therapeutic indications for propofol state that “propofol should be used only for induction and maintenance of general anesthesia and sedation of ventilated patients receiving intensive care.” A few reports have focused on the efficacy of propofol for lengthy procedures, such as endoscopic retrograde cholangiopancreatography (ERCP), endoscopic ultrasonography, and ESD [10, 18–20]. However, to the best of our knowledge, a study of the specific method of propofol administration for therapeutic endoscopy under deep sedation with spontaneous respiration has not been reported.

We analyzed our experience with propofol anesthesia during therapeutic endoscopy and established an optimal method of administration of propofol for this purpose.

## 2. Methodology

This study was conducted in accordance with the Declaration of Helsinki and was approved by the Institutional Review Boards of the authors' institutions.

### 2.1. Patients

All procedures were recorded in prospective endoscopy databases on patients who underwent endoscopic resection. The databases were examined to identify all patients who underwent ESD or endoscopic mucosal resection (EMR) of the esophagus and stomach under anesthesia with 1.0% propofol from April 2012 to April 2013. This study included 89 patients (67 males and 22 females; mean age 71.2 y; 12 esophagus and 77 stomach) (Table 1).

Two experienced therapeutic endoscopists performed the procedures. Patients received supplemental oxygen (2 L/min) by nasal cannula in the endoscopic room as their vital signs and oxygen saturation were continuously monitored using a standard 3-lead electrocardiogram, pulse oximetry, and automatic blood pressure equipment. All patients received an intravenous maintenance electrolyte solution (Na^+^: 35 mEq/L, K^+^: 20 mEq/L, Cl^−^: 35 mEq/L, and L-Lactate^−^: 20 mEq/L) at 80 mL/h before and during the procedure. All medications were administered by physicians from the endoscopy division who did not participate in the actual endoscopic procedures. At least one physician with advanced training in basic and cardiac life support was present during every procedure. An anesthesiologist was also on standby in case of an emergency, and resuscitation equipment was always present in the endoscopic room.

### 2.2. Propofol Administration

**Figure 1:**
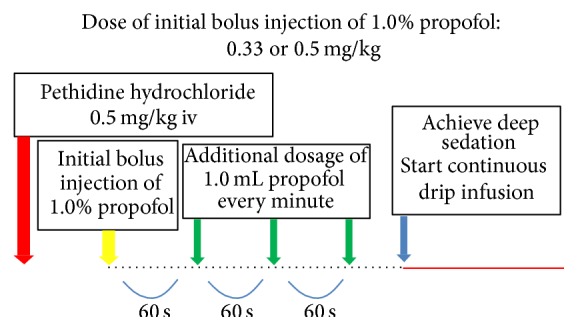
Introduction of sedation: (1) bolus injection of pethidine hydrochloride (0.5 mg/kg) was administered; (2) initial dose of 1.0% propofol was administered; (3) 1.0 mL of propofol was added every min until anesthesia to level 6 on the Ramsey Sedation Scale (patient exhibits no response) was achieved.

**Figure 2:**
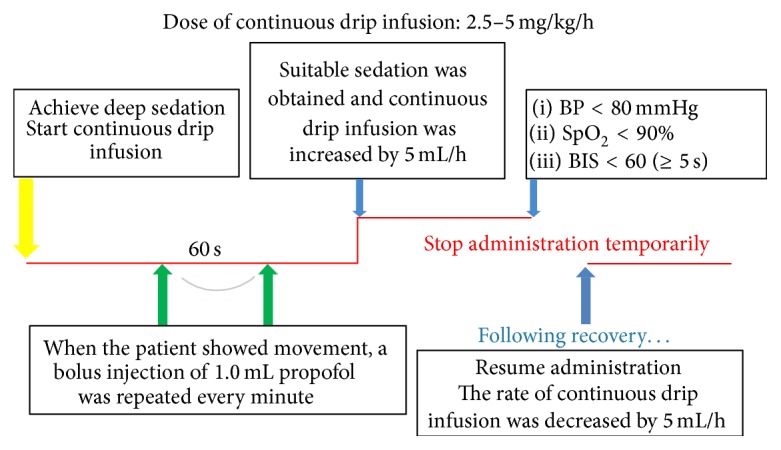
Maintenance of sedation. (1) Continuous drip infusion was performed using an automatic infusion pump (atom syringe pump S-1235; Atom Medical Corp.). (2) When the patient moved, a bolus injection of 1.0 mL propofol was repeated every min until suitable sedation was obtained. The infusion rate was increased by 5 mL/h. (3) Continuous drip infusion was stopped temporarily under the following conditions: SpO_2_ < 90%, systolic blood pressure (SBP) < 80 mmHg, or BIS score < 60 (≥5 s). (4) Following recovery, the infusion rate was decreased by 5 mL/h.

Introduction and maintenance doses of propofol that were examined were changed based on efficacy and/or adverse events and classified retrospectively into 5 periods (Table 2). After the beginning of Period 1, when it was deemed that the dose of propofol was insufficient or excessive, another dosage was selected for examination and the next period was begun but with a different group of patients. In all dosage periods, the initial dose of 1.0% propofol was administered after bolus injection of pethidine hydrochloride (0.5 mg/kg), and 1.0 mL of propofol was added every min until the patient fell asleep, as determined by the Ramsey Sedation Scale 6 (Figure 1). Subsequently, continuous drip infusion using an automatic infusion pump (atom syringe pump S-1235; Atom Medical Corp., Tokyo, Japan) was performed to maintain the depth of sedation. When the patient showed movement, a bolus injection of 1.0 mL propofol was repeated every min until suitable sedation was obtained, and the infusion rate was increased by 5 mL/h regardless of the patient's body weight. Continuous drip infusion was stopped temporarily under the following conditions: SpO_2_ < 90%, systolic blood pressure (SBP) < 80 mmHg, or BIS score < 60 (≥5 s). Following recovery, the infusion rate was decreased by 5 mL/h regardless of the patient's body weight (Figure 2). Oxygen saturation (SpO_2_; under oxygenation at a rate of 2 L/min) and blood pressure were monitored during all procedures.

### 2.3. Bispectral Index Setting

BIS scores were monitored using a BIS monitor (BIS VISTA Monitoring System, Covidien, Mansfield, MA, USA) and a specific BIS Quatro Sensor (Covidien) at only Period 5. The target range for the BIS monitoring index was 60 to 80.

We calculated the dose of propofol at the time of introduction and, during the maintenance phase, the number of additional bolus injections in the introduction phase, increases and decreases in maintenance doses, and the average total dose of propofol. The incidence of cardiorespiratory suppression was evaluated for each period. The incidence of a BIS score <60 was also evaluated in Period 5.

### 2.4. Statistical Analysis

One-way analysis of variance (ANOVA) was used to evaluate patients' background, and Fisher's exact test was used to evaluate the incidence of adverse events during each period. A *P* value of <0.05 was considered significant. All statistical analyses were performed using SAS_ version 8.2 (SAS Institute, Cary, NC, USA).

## 3. Results

During introduction, no cardiorespiratory suppression occurred in any of the periods. During maintenance, hypotension (SBP < 80 mmHg) occurred in 11 (40.7%) patients at Period 1 (introduction dose : maintenance dose; 0.5 mg/kg : 5 mg/kg/h, *n* = 27). When we moved to examining Period 2 (*n* = 11), the dosage for the combination of introduction : maintenance was decreased to 0.33 mg/kg : 3.3 mg/kg/h compared with that for Period 1. Hypotension was reduced to affect only 3 of the 11 patients (27.3%). However, the number of additional bolus injections at the introductory phase was increased. At Period 3 (*n* = 7), the dose combination was increased to 0.5 mg/kg : 3.3 mg/kg/h. The number of additional bolus injections was decreased. However, hypotension occurred in 3 of the 7 patients (42.9%), and the dose combination was reduced to 0.5 mg/kg : 2.5 mg/kg/h at Period 4 (*n* = 14). Thereafter, hypotension was attenuated and observed in only 4 of the 14 patients (28.6%). The same dose combination was employed with the BIS monitor at Period 5 (*n* = 30) and hypotension was attenuated and observed in only 3 of the 30 patients (10.0%). Comparison of the incidence of adverse events between Period 1 and Periods 2–4 showed no significant differences. However, the rate of adverse events for Period 5 was significantly lower than that for Period 1 (*P* = 0.012). Hypotension had been resolved quickly in all patients by stopping the propofol administration for a few minutes and by temporarily increasing the drip rate of the infusion with no need for further intervention and discontinuation of the procedure. In contrast, no respiratory suppression (SpO_2_ < 90%) occurred during the maintenance phase in any period.

## 4. Discussion

Propofol is being used in some institutions in Japan for therapeutic endoscopy. However, the method of administration differs according to the facilities and doctors and has not been clearly established. We established the (1) dose for introduction, (2) method of additional bolus injections in the introduction phase, (3) maintenance dose, and (4) criteria for increases and decreases in the maintenance dose. We then selected and examined various dosages and methods of administration to determine a simple and safe method of propofol administration for therapeutic endoscopy.

The safety instructions provided by the manufacturer of propofol provided that propofol should be titrated (0.5 mg/kg every 10 s for an adult) according to the patient's response until clinical signs show the onset of anesthesia. For the introduction of general anesthesia, most adult patients are likely to require between 2.0 and 2.5 mg/kg of propofol. The required rate of administration is 4 to 10 mg/kg/h in the maintenance phase. Based on this, for endoscopy we reduced the initial introduction dose and maintenance dose to be examined to provide a safety margin in consultation with the anesthesiologist at our hospital (Period 1, introduction dose : maintenance dose = 0.5 mg/kg : 5 mg/kg/h).

It is well known that propofol may induce arterial hypotension and respiratory depression. As the cause, the effect of sympathetic nervous system inhibition was pointed out [21–23], and a direct vasodilatation effect was reported regarding arterial hypotension [24–26]. In the report using a similar sedation method with propofol continuous intravenous infusion, Kiriyama et al., to decrease the amount of propofol at the maintenance phase, used hypotension (SBP < 80 mmHg) and oxygen desaturation (SpO_2_ < 90%), as indicators for the decrease in criteria and not the heart rate. Yamagata et al. did not specify the criteria for decreasing the dosage of propofol at the maintenance phase [27]. It is known that changes in the heart rate, particularly tachycardia, often occur as a reflection of hypotension. Therefore, we used hypotension (SBP < 80 mmHg) and oxygen desaturation (SpO_2_ < 90%) as indicators of an adverse event based on previous reports. With regard to the patients' background, particularly the ASA physical status and a medical history of coronary heart disease, there was no significant difference between periods.

Hypotension (SBP < 80 mmHg) occurred in 11 (40.7%) patients at Period 1 (*n* = 27). It was deemed that the dose of propofol was excessive; therefore, we moved to Period 2 (*n* = 11) wherein the introduction : maintenance dose was reduced to 0.33 mg/kg : 3.3 mg/kg/h. The rate of hypotension was reduced and occurred in only 3 of 11 (27.3%) patients. However, the number of additional bolus injections at the introductory phase was increased, which was not desirable. At Period 3 (*n* = 7), the dose combination was increased to 0.5 mg/kg : 3.3 mg/kg/h and the number of additional bolus injections was decreased, but the rate of hypotension increased and occurred in 3 of the 7 (42.9%) patients. Using a method of administration similar to that in Period 3, Kiriyama et al. reported that adverse events such as hypotension and respiratory suppression occurred in 0% of patients [20]. In comparison with Period 3 and that report, no significant difference was seen in the patients' backgrounds. The reason for the high frequency of hypotension in our examination was not clear, and we moved to the next period. The dose combination for introduction : maintenance was reduced to 0.5 mg/kg : 2.5 mg/kg/h in Period 4 (*n* = 14). Thereafter, hypotension was attenuated and observed in only 4 of the 14 (28.6%) patients. We attempted to reduce the rate of hypotension further and thought that the ability to minimize the administered dose of propofol by using BIS monitoring may decrease the incidences of adverse events caused by oversedation. Therefore, at Period 5 (*n* = 30), a BIS monitor was employed and the same dosage as for Period 4 was examined. As a result, hypotension was attenuated to 3 of the 30 (10.0%) patients and the rate of adverse events for Period 5 was significantly lower than that for Period 1 (*P* = 0.012).

A BIS monitor can objectively evaluate the depth of sedation by monitoring brain waves. BIS value is a dimensionless number scaled from 100 to 0, with 100 representing an awake electroencephalograph and 0 representing complete electrical silence (cortical suppression). BIS values of 65–85 have been recommended for sedation, whereas values of 40–65 have been recommended for general anesthesia [28]. There is a report that BIS values of 76–85 have been recommended for moderate sedation [29, 30]. A higher level of sedation is required for therapeutic endoscopy compared with conventional endoscopy. Therefore, we set the BIS range at 60–80.

A previous study showed that BIS monitoring led to a reduction in the mean propofol dose when the BIS value was used as the primary target for sedation in ERCP procedures [31]. Monitoring with BIS during ESD procedures led to higher satisfaction scores from patients and endoscopists [32]. However, to the best of our knowledge, there is no report that BIS monitoring led to a reduction in adverse events such as hypotension and respiratory suppression.

A direct correlation between the BIS and hypotension has not been shown. It is well known that hypotension and respiratory depression are caused by oversedation. BIS is correlated with depth of sedation, and there is a possibility that hypotension caused by oversedation is captured by a change in BIS. In our study, propofol doses in Period 4 and Period 5 were the same. However, the incidence rate of hypotension was decreased by employing a BIS monitor at Period 5.

We decreased the maintenance dose of propofol when the BIS value was lower than 60 in Period 5. A BIS monitor can continuously detect a numerical change. Therefore, there was a possibility that a slight change in the BIS value could be sensed before a serious circulatory depression occurred and could decrease the dose of propofol sooner, thus preventing hypotension. Although further examination will be needed, the results of the present study indicated the possibility that BIS monitoring might be useful in preventing adverse events such as hypotension.

The procedure did not need to be discontinued in any of the patients studied. At Period 5, data showed that a safe and stable sedation could be maintained. This suggested that our dosage and the BIS setting for propofol administration were suitable for therapeutic endoscopy. However, a few cases required a frequent increase in dosage because of insufficient sedation. Among Period 5 patients, 2 patients required more than 10 additional bolus injections at the introductory phase and 5 patients required more than 4 increases in maintenance doses. To better understand background factors in such cases that would necessitate frequent increases in dosage, we are now performing a prospective study of the same method as in Period 5. This study is registered with the UMIN Clinical Trial Registry (UMIN000010547) in Japan.

The present study had some limitations. Hemodynamic data were insufficient (e.g., heart rate, diastolic and mean arterial pressure, and left ventricular function). The number of patients included was small. As it was a retrospective study, further randomized prospective studies of adjustment of administration and monitoring techniques conducted in a large population are necessary for optimal propofol administration for therapeutic endoscopy.

## 5. Conclusions

This newly developed propofol anesthesia with BIS monitoring protocol (Period 5) might be safe and useful for therapeutic endoscopy under deep sedation with spontaneous respiration.

## Figures and Tables

**Table 1:** Characteristics of study patients.

	Period 1	Period 2	Period 3	Period 4	Period 5	Total	*P* value
Male/female, *n*	22/5	8/3	5/2	9/5	23/7	67/22	0.76
Age, mean ± SD, years	71.0 ± 7.3	72.9 ± 6.6	70.9 ± 11.2	72.9 ± 6.5	70.9 ± 7.5	71.2 ± 7.6	0.81
Esophagus/stomach, *n*	3/24	1/10	1/6	2/12	5/25	12/77	0.97
Body weight, mean ± SD, kg	61.1 ± 8.8	62.9 ± 9.7	58.2 ± 9.5	55.2 ± 9.2	61.1 ± 11.6	60.2 ± 10.2	0.32
BMI, mean ± SD, kg/m^2^	23.1 ± 2.0	23.5 ± 3.1	22.8 ± 3.6	22.0 ± 3.5	23.2 ± 3.2	23.0 ± 3.0	0.75
ASA physical status, *n* (%)							0.26
ASA I	7 (25.9)	4 (36.4)	2 (28.6)	2 (14.3)	4 (13.3)	19 (21.3)	
ASA II	19 (70.3)	6 (54.5)	5 (71.4)	10 (71.4)	21 (70.0)	61 (68.5)	
ASA III	1 (3.8)	1 (9.1)	0	2 (14.3)	5 (16.7)	9 (10.2)	
Past history of CHD, *n* (%)	1 (3.8)	1 (9.1)	0	1 (7.2)	4 (13.3)	7 (7.9)	0.65

BMI: body mass index, ASA: American Society of Anesthesiologists, and CHD: coronary heart disease.

**Table 2:** Propofol dosages and the incidence of adverse events during each period of the study.

	Period 1 (*n* = 27)	Period 2 (*n* = 11)	Period 3 (*n* = 7)	Period 4 (*n* = 14)	Period 5 (*n* = 30)
Propofol dosing					
Dose of initial bolus injection, mg/kg	0.5	0.33	0.5	0.5	0.5
Dose of continuous drip infusion, mg/kg/hr	5	3.3	3.3	2.5	2.5
Average number of additional bolus injections at introductory phase, (range)	1.07 (0~6)	4.0 (1~13)	1.6 (0~5)	3.8 (0~7)	3.6 (0~17)
Average number of increasing maintenance doses, (range)	0.7 (0~5)	1.5 (0~2)	1.3 (0~5)	2.6 (1~5)	1.9 (0~7)
Average number of decreasing maintenance doses, (range)	0.63 (0~3)	0.18 (0~1)	0.86 (0~2)	0.36 (0~2)	0.17 (0~2)
Total propofol dose, mean ± SD, mL	48.5 ± 22.6	60.1 ± 28.9	49.6 ± 41.9	44.4 ± 23.0	52.1 ± 35.7
Procedure time, mean ± SD, min	105.6 ± 39.8	126.5 ± 42.8	106.7 ± 65.4	103.9 ± 57.9	115.9 ± 75.5
Incidence of adverse events, *n* (%)					
SpO_2_ <90%	0	0	0	0	0
Systolic blood pressure <80 mm Hg	11 (40.7)	3 (27.3)	3 (42.9)	4 (28.6)	3 (10.0)^*∗*^
BIS score < 60 (>5 s)	NA	NA	NA	NA	1 (3.3)
Discontinuation of procedure	0	0	0	0	0

^*∗*^ *P* = 0.012; Period 1 versus Period 5.
